# Navigating the Complexities of Implementing Evidence‐Based Respiratory Muscle Training in Post‐Mechanical Ventilation Care: A Qualitative Study

**DOI:** 10.1002/nop2.70408

**Published:** 2025-12-23

**Authors:** Jianing Yin, Xiaomin Guan, Qi Zhang, Yan Shen, Xiaodan Zhang, Lan Chen

**Affiliations:** ^1^ Department of Nursing Hangzhou First People's Hospital Hangzhou China; ^2^ Department of Nursing Shanghai General Hospital Shanghai China

**Keywords:** airway extubation, barriers, facilitators, intensive care units, mechanical ventilation, qualitative, respiratory muscle training

## Abstract

**Aim:**

This study aimed to explore the perceptions of medical personnel and patients regarding respiratory muscle training (RMT) for patients after mechanical ventilation withdrawal.

**Design:**

A qualitative study.

**Methods:**

Semi‐structured interviews were conducted with 12 medical staff and 9 eligible patients between August and December 2023. Guided by the integrated Promoting Action on Research Implementation in Health Services (i‐PARIHS) framework, the interviews focused on facilitators and barriers to RMT. Data were coded according to the i‐PARIHS domains (context, innovation and recipients) and analysed thematically.

**Results:**

Three main themes emerged: (1) recipients—encompassing medical staff attitudes, knowledge training, patient physical factors, and willingness to engage in RMT, (2) context—including interdisciplinary collaboration, quality control, human resources, incentives, training facilities, leadership focus and the role of respiratory therapists and (3) innovation—highlighting the advantages of RMT and challenges in the continuity of evidence‐based application.

**Conclusion:**

This study identified key barriers and facilitators to respiratory muscle training in post‐mechanical ventilation care, providing an evidence base for developing intervention strategies in the future.

## 1 Introduction and Background

Mechanical ventilation (MV) is a common intervention for critically ill patients in intensive care units (ICUs) (Ma et al. 2022). In severe cases, MV is often accompanied by sedation and immobilisation, leading to prolonged disuse of the respiratory muscles and subsequent muscle weakness (Minhas et al. 2015). Inspiratory muscle weakness may result in hypoventilation, dyspnoea, reduced exercise tolerance and respiratory failure, while expiratory muscle weakness impairs cough reflex efficiency, hindering the clearance of respiratory secretions (Ma et al. 2022; Vorona 2018). Notably, respiratory muscle weakness, particularly in the diaphragm, occurs twice as frequently as limb muscle weakness (Dres et al. 2017) and is associated with poor prognosis, prolonged MV, increased reintubation rates, higher risk of ventilator‐associated pneumonia, extended ICU stays, elevated morbidity and mortality and increased hospital readmission rates (Adler et al. 2014; Medrinal et al. 2016).

RMT is an intervention aimed at enhancing the strength and endurance of inspiratory and/or expiratory muscles to improve respiratory function and facilitate recovery in patients following prolonged MV (Hearn et al. 2022; Watson et al. 2022). With the growing emphasis on early rehabilitation for critically ill patients, RMT has emerged as a promising therapy for mechanically ventilated patients in the ICU (Patsaki et al. 2024). This targeted training requires a multidisciplinary approach, involving collaboration among medical, nursing and physiotherapy teams to reduce sedation, optimise patient alertness and enable active participation in breathing exercises (Bissett et al. 2020, 2019). RMT can be delivered using mechanical (spring‐loaded) or electronic devices that provide adjustable inspiratory loads (Bissett et al. 2020). Evidence suggests that RMT improves inspiratory muscle strength (Bissett et al. 2016; Martin et al. 2011; Tonella et al. 2017; Vorona 2018) enhances quality of life (QOL) (Bissett et al. 2016), and may improve weaning outcomes (Cader et al. 2010; Elkins and Dentice 2015; Martin et al. 2011; Tonella et al. 2017). Despite its benefits, RMT is often underutilised due to several barriers. Patient‐related factors, such as safety concerns and readiness, are significant challenges (Liew et al. 2021). Additionally, many nurses express concerns about exacerbating patient injuries, potentially due to insufficient knowledge of safe RMT practices (Parry et al. 2017). Institutional barriers, including staffing shortages, inadequate equipment (Anekwe et al. 2019; Goddard et al. 2018) and limited familiarity with RMT protocols (21), further hinder its implementation. Addressing these barriers is essential to promote the effective use of RMT in ICU settings.

The significant role of MV in the ICU is met with the equally critical challenge of supporting patients through the weaning process—a stage in which RMT becomes paramount. However, implementing RMT effectively is not straightforward, as its application is profoundly shaped by cultural and contextual factors specific to each ICU setting. For instance, studies conducted in Asian ICUs have revealed low adherence to RMT protocols, with therapists frequently reporting infrequent use and substantial implementation barriers (Major et al. 2024; Yin et al. 2024). Although RMT is more established in Western ICU contexts, it remains far from standardised or uniformly effective. Its application varies widely depending on differences in patient populations, workplace cultures and ICU structures across regions (Braithwaite et al. 2017). This variability underscores a broader and persistent challenge in healthcare: the difficulty of integrating evidence‐based practices into routine care, which often limits their sustained translation into clinical practice (Curtis et al. 2017). This gap between evidence and practice is a well‐documented phenomenon in implementation science. Research suggests it arises from a complex interplay of individual, organisational and systemic barriers—such as lack of knowledge, negative attitudes, resource constraints, and insufficient organisational support (Mensiková et al. 2022; Zhou et al. 2023). These can include lack of knowledge, negative attitudes, insufficient time or resources and inadequate organisational support or leadership. It is precisely this gap between the theoretical benefits of RMT and its inconsistent real‐world application that highlights the necessity of the present study. By systematically identifying the context‐specific barriers and facilitators influencing RMT implementation, this research aims to inform the development of tailored strategies to optimise rehabilitation for patients following MV withdrawal.

## 2 Methods

### 2.1 Study Design

This qualitative study explored the barriers and facilitators to implementing RMT in clinical physiotherapy practice for patients after MV withdrawal. Using a descriptive qualitative design, semi‐structured interviews were conducted in accordance with the Standards for Reporting Qualitative Research (SRQR) (O'Brien et al. 2014).

### 2.2 Theoretical Framework

This study was guided by the integrated Promoting Action on Research Implementation in Health Services (i‐PARIHS) framework (Gustavson et al. 2023; Harvey and Kitson 2016). The framework posits facilitation as a central element for aligning innovation, recipients and context to enable successful implementation. It examines the dynamic interactions among three core domains: (1) Innovation, defined by intervention characteristics such as complexity, relative advantage over existing practices, usability, and supporting evidence (e.g., research, clinical expertise and patient preferences); (2) Recipients, encompassing individuals impacted by the innovation, including patients and care providers; and (3) Context, which incorporates internal organisational factors (e.g., leadership support, culture, priorities, evaluation processes and learning networks) as well as external factors (e.g., policy drivers, incentives, mandates and inter‐organisational collaborations) (Gustavson et al. 2023; Harvey and Kitson 2016). This framework informed the development of the interview guides, data collection and the subsequent analysis, providing a consistent lens through which implementation factors were explored.

### 2.3 Study Setting

This study was conducted in a 52‐bed adult ICU within a 1550‐bed Chinese tertiary hospital. The ICU provides comprehensive intensive care services.

### 2.4 Sample Study Setting

Purposive sampling was employed to recruit patients and ICU staff (including doctors, respiratory therapists and nurses) between August and December 2023.

Patients were included if they met the following criteria: (1) age ≥ 18 years, (2) withdrawal from MV after ≥ 72 h, (3) ability to communicate effectively; and (4) voluntary participation. Exclusion criteria included: (1) unconsciousness or mental disorders; and (2) extreme hemodynamic instability or frequent malignant arrhythmias, rendering them unable to cooperate with the interview.

ICU staff were eligible if they: (1) had over 2 years of ICU clinical or nursing experience; (2) demonstrated strong verbal communication skills; and (3) provided informed consent for voluntary participation. Staff on probationary periods were excluded.

### 2.5 Data Collection

Semi‐structured interviews were conducted for data collection, guided by the i‐PARIHS framework to develop the interview guide (Harvey and Kitson 2016). Two versions of the interview outline were created. Medical staff version: (1) Please describe the implementation of RMT for patients withdrawn from MV in the ICU. What challenges have been encountered, and what are the perceived causes of these challenges? (2) What changes have you observed before and after implementing RMT? Do you believe these changes can be sustained in your ward long‐term? (3) Which organisational factors (e.g., culture, resources, leadership) influence the implementation of RMT? (4) Which personnel impact the implementation of RMT, and in what ways? Patient version: (1) Please describe your experiences and feelings during RMT. (2) What factors influenced your participation in RMT? (3) What RMT activities are you currently performing? Follow‐up questions were asked to explore reasons for any untrained programs. Data were reviewed and analysed iteratively during collection, with interview guides adapted as necessary to ensure depth and relevance.

A purposive sampling strategy was employed to recruit ICU staff participants to ensure a diversity of perspectives, roles and clinical experience. Potential participants, including intensivists, respiratory therapists and critical care nurses, were identified in collaboration with the unit manager. They were provided with study information and invited to participate. Semi‐structured interviews were conducted by the primary researcher at a time convenient for the participant. Interviews were scheduled either before, after, or during the participant's shift, depending on unit activity and staff preference, to minimise disruption to clinical care. All interviews were conducted in a private room within or near the ICU to ensure confidentiality. The interviews lasted about 30 min.

The interview durations with patients ranged from approximately 20–40 min. The flexible approach was necessary to accommodate individual patient recovery status and prevent fatigue.

### 2.6 Data Analysis

A rapid qualitative, deductive team‐based approach was utilised, informed by directed content analysis (Gustavson et al. 2023). Directed content analysis was conducted using the i‐PARIHS framework (Harvey and Kitson 2016) to guide data collection, analysis and interpretation (Hsieh and Shannon 2005). Four research team members held bi‐weekly meetings to analyse the data, categorising raw data into predefined themes based on timepoints (before and after implementation) and identifying representative excerpts to illustrate these themes. The multidisciplinary team also engaged in discussions to compare themes across timepoints. Data saturation was determined by the absence of new or emerging themes (Saunders et al. 2018).

### 2.7 Rigour

This study followed Guba and Lincoln's quality model as illustrated in (Nowell et al. 2017) to ensure rigour. First, to achieve credibility, we used participant collaboration and sample saturation principle. Second, maximum variation purposeful sampling and thick description were used to ensure transferability. Third, during the stages of data collection and data analysis, field notes and analysis memos were used to record the researchers’ actions, decisions and continuous reflection, which helped establish the confirmability of the study. Fourth, dependability was ensured by using the Reporting Qualitative Research (COREQ) checklist (Nowell et al. 2017).

### 2.8 Ethical Considerations

Ethics approval was granted by the hospital (No.【2023】472). All participants were asked to sign a consent form after having read the participant information sheet. Participant demographic data was summarised as group data to protect their privacy.

## 3 Findings

This study included 21 participants: 9 patients, 8 nursing staff, 3 doctors and 1 respiratory therapist. Demographic characteristics of patients and medical staff are detailed in Tables 1 and 2, respectively. Primary themes were identified for each i‐PARIHS construct: Recipients: Themes included medical staff attitudes, knowledge and training, patient physical factors and willingness to engage in training. Context: Themes focused on interdisciplinary collaboration, quality control, human resources, incentives, training facilities, leadership focus and the role of respiratory therapists. Innovation: Themes highlighted the benefits of innovation and challenges in sustaining evidence‐based practices. Representative excerpts from field notes are presented in Table 3.

**TABLE 1:** Patient characteristics.

Variable	Outcome
Sex	
Male (*n*, %)	5 (55.6)
Female (*n*, %)	4 (44.4)
Age in years (median, IQR)	56 (30)
MV in days (median, IQR)	6 (3)

**TABLE 2:** Medical staff characteristics.

Variable	Nurses	Doctors and respiratory therapist
Total	8	4
Sex		
Male (*n*, %)	0 (0)	2 (50.0)
Female (*n*, %)	8 (100)	2 (50.0)
Work experience in ICU, years (median, IQR)	11 (11)	6.5 (12)
Education		
Junior college	2 (25)	0 (0)
Bachelor's degree (*n*, %)	6 (75)	1 (25)
Master's degree (*n*, %)	0 (0)	2 (50)
PhD degree (*n*, %)	0 (0)	1 (25)

**TABLE 3:** Themes and sub‐themes.

Themes	Sub‐themes
Recipients	Attitude of medical staff
	Knowledge and training
	Patient physical factors
	Patient's willingness to train
Context	Interdisciplinary collaboration
	Quality control
	Human resources and incentives
	Training facilities
	Leadership focus
	Respiratory therapist
Innovation	Advantages of innovation
	Insufficient continuity in the application of evidence

### 3.1 Theme 1. Recipients

#### 3.1.1 Attitude of Medical Staff

Some healthcare professionals perceive that ICU patients primarily receive treatment and rehabilitation, which hinders the advancement of RMT for patients after MV withdrawal.

N: ‘I believe RMT can be promoted, but it currently lacks sufficient attention. Although early rehabilitation is emphasized, the focus for critically ill patients remains predominantly on treatment. Managing patients with varying severity often diverts attention and resources toward urgent medical needs, leaving limited capacity for rehabilitation. In my view, the primary role of the ICU is treatment, while rehabilitation tasks should be addressed in general wards.’

#### 3.1.2 Knowledge and Training

Interviews revealed that nurses lack standardised training in RMT, with some relying on personal experience to guide patients in RMT practices.

N: ‘Nurses lack specialized training in this area, as there is no formal training program. Instead, expertise is developed through clinical experience and accumulated practical knowledge.’

D: ‘We lack systematic training in this area, as it is a highly specialized field, whether in the US or Australia. Our theoretical knowledge is insufficient and we are unfamiliar with the use of certain equipment due to the absence of specialized training.’

#### 3.1.3 Patient Physical Factors

Most interviewees highlighted that patients’ physical conditions—such as pain, weakness, incapacitation and unconsciousness—limited their ability to engage in RMT after mechanical ventilation withdrawal.

P: ‘Initially, I lacked the strength to continue the exercises, and the effort felt overwhelming. […] The training causes significant pain at the wound site, outweighing its perceived benefits. […] With multiple tubes in place, I avoided movement due to fear of dislodgement and increased pain.’

N: ‘When experiencing pain, patients are often unable to perform the exercises, not unwilling. Many avoid deep breathing due to pain, despite their desire to participate. […] Prolonged use of anesthetics and muscle relaxants leaves patients with insufficient strength to engage in training.’

D: ‘Some patients are very weak after extubation, and others are in excruciating pain after surgery and their attention is not focused on it. […] Training is also more difficult for patients with abdominal surgery or systemic infections.’

#### 3.1.4 Patient's Willingness to Train

When patients recognise that RMT aids rehabilitation and shortens ICU stays, they are more likely to actively cooperate with healthcare staff and demonstrate greater initiative.

P: ‘I know that if I do it properly, I won't have to put the tube back in, the tube in my throat is just too much, so I'm going to do it carefully. […] Doing well means I can return to the ward sooner and reunite with my family. […] The nurses explained that these exercises aid recovery, so I am committed to performing them diligently—if they recommend 10 repetitions, I will do 20.’

N: ‘Most patients are willing to cooperate, driven by the goal of reducing ICU stays and transitioning to a general ward to reunite with their families.’

P: ‘I'm old, I cannot recover as quickly as younger individuals. […] During the morning check‐in, the director commended my progress, which motivated me to perform the exercises more diligently.’

### 3.2 Theme 2. Context

#### 3.2.1 Interdisciplinary Collaboration

Currently, the ICU lacks a dedicated multidisciplinary rehabilitation team focused on providing RMT for patients after MV withdrawal.

D: ‘It would be ideal to establish a dedicated team for patient rehabilitation or RMT. […] Currently, the ICU lacks a standardized rehabilitation team.’

Additionally, unclear division of responsibilities among healthcare professionals resulted in limited engagement.

N: ‘Nurses vary in their skill levels and perceptions, with some assuming that RMT falls under the respiratory therapist's role. With respiratory therapists now guiding patients, nurses may perceive this as outside their responsibilities and disengage. […] Effective RMT implementation requires multidisciplinary collaboration, but the current division of responsibilities within the healthcare team remains unclear.’

#### 3.2.2 Quality Control

Challenges include the lack of a standardised implementation process and dedicated staff to oversee the training, leading to disorganisation and disruption of clinical workflows.

N: ‘A dedicated system, including standardized processes and guidelines, should be established for this patient population. […] A designated nurse could oversee the training implementation across wards to ensure consistency and quality.’

D: ‘A standardized process should be developed to determine which patients require RMT, along with the specific content and intensity of the training for each day.’

#### 3.2.3 Human Resources and Incentives

Conducting RMT is time‐intensive, adding to the workload of healthcare workers, and extending it to all patients would further strain their capacity.

N: ‘Our workload is particularly heavy, as we assist respiratory therapists with these exercises while prioritizing therapeutic care over RMT. Each session is limited to about 10 min due to time constraints. Manpower is insufficient, especially during busy periods when even four staff members struggle to manage therapeutic care. […] I conduct RMT when time permits, but during emergencies or high workloads, it becomes impossible. With only two respiratory therapists available, who are often required in the A&E ward, the demand exceeds capacity.’

D: ‘Our limited manpower restricts cardiopulmonary rehabilitation to a secondary role, performed only after completing primary duties. While other departments have been supportive, resources remain inadequate, particularly due to the shortage of rehabilitation specialists.’

Additionally, the absence of incentives for healthcare workers reduces their motivation, further hindering the implementation of RMT.

N: ‘Currently, lower income significantly impacts motivation. […] With scarce human resources and a low nurse‐to‐patient ratio, performance distribution issues further demotivate staff. A fair and reasonable performance allocation could provide some incentive.’

#### 3.2.4 Training Facilities

The lack of relevant professional instruments and equipment also limits RMT to a certain extent.

N: ‘The department currently has only one portable lung function instrument, and respiratory function trainers are not widely used. […] Families are not routinely asked to purchase these trainers due to their high cost. […] While respiratory function trainers are beneficial, the requirement for families to purchase them limits accessibility and convenience.’

D: ‘Those training devices we have are also insufficient.’

#### 3.2.5 Leadership Focus

Leadership support improves resources. Including respiratory therapists enhances RMT and training outcomes.

N: ‘Our director has recruited two respiratory therapists and prioritizes patient respiratory rehabilitation. […] He remains highly attentive to this area, consistently urging us to initiate cardiopulmonary rehabilitation as early as possible.’

#### 3.2.6 Respiratory Therapist

The involvement of respiratory therapists makes RMT more specialised, assessing patients and guiding respiratory muscle training.

N: ‘Under the guidance of respiratory therapists, our practices have become more specialized. They also remind nurses to prioritize RMT after patient extubation. […] A key advantage in our ICU is the presence of two dedicated respiratory therapists.’

P: ‘The respiratory therapists provide guidance and correct my techniques when errors occur, demonstrating a high level of professionalism. […] They actively demonstrate proper methods, showcasing their expertise.’

### 3.3 Theme 3. Innovation

#### 3.3.1 Advantages of Innovation

RMT can enhance patient recovery, reduce ICU length of stay, improve ICU turnover rates, and demonstrate the professional value of nursing staff.

N: ‘Patients often lack strength after mechanical ventilation withdrawal. Previously, we primarily advised them to perform basic deep breathing exercises, which yielded limited recovery outcomes. Now, with systematic RMT, the improvement in respiratory muscle strength is more pronounced, and recovery progresses faster than before.’

N: ‘When guiding patients through training, I feel more confident than before. If patients have questions, I explain that the exercises are evidence‐based, derived from guidelines and literature with high levels of evidence. This approach has increased patient trust and reinforced the value of our work.’

D: ‘This evidence, derived from high‐quality literature such as guidelines, is highly beneficial for patients. […] Using this evidence to guide RMT has proven effective, with patients demonstrating significant recovery post‐training.’

#### 3.3.2 Insufficient Continuity in the Application of Evidence

During the evidence‐based practice project, nurse compliance improved through supervision and repeated emphasis by implementers. However, due to heavy clinical workloads and the critical condition of patients, rehabilitation training was often deprioritized.

N7: ‘RMT for patients after mechanical ventilation withdrawal is not a routine practice in our department and is only being implemented as part of a recent project. Although department leaders emphasize its importance, the critical condition of recently admitted patients, frequent resuscitations, and heavy workloads have limited its consistent application. […] With patient acuity high and routine treatments demanding priority, RMT is often overlooked despite ongoing reminders, as lifesaving interventions take precedence.’

## 4 Discussion

This study employed qualitative methods, guided by the i‐PARIHS framework, to explore the potential, barriers and facilitators for implementing RMT in clinical physiotherapy practice for patients after MV withdrawal.

In the context of a healthcare paradigm shift, multidisciplinary cooperation has become essential, with collaborative networks playing a critical role in practice change. A key finding from our analysis was the critical gap in multidisciplinary collaboration for RMT implementation. Specifically, our results indicate that the absence of a dedicated rehabilitation team and unclear role definitions among healthcare professionals significantly hindered RMT adoption in the ICU setting. This finding aligns with the Recipients domain of the i‐PARIHS framework, highlighting how individual and team‐level factors influence implementation success. Mukpradab et al. (Mukpradab et al. 2022) emphasised that effective multidisciplinary collaboration requires clearly defined responsibilities to facilitate training implementation. Our findings reinforce and contextualise this perspective by providing concrete evidence of how role ambiguity and the lack of a coordinated team structure directly impact RMT delivery in the specific context of post‐ventilator care. To address these challenges, a multidisciplinary team comprising doctors, nurses and respiratory therapists should be established in the ICU. Responsibilities among healthcare professionals must be clarified, and the distribution of RMT tasks actively discussed. Optimal timing for RMT should be determined, interprofessional communication improved and dedicated RMT nurses integrated into routine workflows. Furthermore, efforts should be made to secure managerial support in terms of manpower, resources and policies.

Our findings reveal a significant implementation barrier rooted in professional mindset: many healthcare professionals in our setting maintained a traditional ‘treatment–first, rehabilitation–second’ approach to ICU care. This perspective, which prioritises acute medical management over proactive rehabilitation, directly hindered engagement with RMT. Within the i‐PARIHS framework, this represents a critical challenge within the Recipients domain, where individual beliefs and attitudes directly shape the adoption of an innovation. However, studies (Bissett et al. 2020) (Bissett et al. 2019) have shown that respiratory rehabilitation during ICU hospitalisation can reduce and prevent functional impairments, improve respiratory, cough and motor functions and accelerate recovery in critically ill patients. Therefore, it is essential to shift the traditional mindset of ‘treatment–first, rehabilitation–second’ and increase awareness of the benefits of RMT among healthcare professionals. Leadership advocacy and supervision can further support RMT implementation alongside this mindset shift. In clinical practice, the role of nurses must be emphasised, with adjustments to integrate nursing‐led rehabilitation training and promote nurse‐driven RMT implementation.

This study revealed that a significant barrier to RMT implementation in our local context is the knowledge gap among nurses, who reported insufficient training in rehabilitation techniques for patients after MV. This finding directly addresses the ‘Recipients’ dimension of the i‐PARIHS framework, highlighting how the characteristics and preparedness of key stakeholders influence implementation success. While international studies show advanced multidisciplinary approaches to RMT in ICU settings (Bissett et al. 2019; Yang et al. 2020), our findings provide important contextual understanding by identifying the specific educational and training needs within our healthcare system. This knowledge gap among frontline staff represents a critical point for intervention. The researcher‐manager‐practitioner collaborative evidence‐based practice model developed by Ge Xiangyu et al. (Ge et al. 2017) emphasises that clinical nurses are the primary drivers of evidence implementation. Promoting evidence translation requires enhancing nurses’ knowledge, adherence and recognition of evidence. Practice departments should enhance training on post‐MV RMT, using managerial influence to improve healthcare professionals’ cognitive behaviours and support evidence‐based nursing. This approach encourages reflection on clinical challenges, boosts confidence in evidence‐based practice and aids research translation. Nurses’ knowledge can also be improved with educational materials like illustrated manuals and instructional videos.

Our study identified several patient‐level physiological barriers to RMT implementation, with patient weakness, sedation‐induced unconsciousness and pain emerging as significant limiting factors, consistent with findings by (Major et al. 2024). These findings directly reflect challenges within the ‘Innovation’ domain of the i‐PARIHS framework, specifically relating to the complexity of delivering RMT to a vulnerable and clinically unstable population. Our results indicate that a standardised, one‐size‐fits‐all approach is ineffective. To address the barrier of patient weakness, our findings suggest a flexible approach of initially reducing the intensity and duration of training, with gradual progression based on tolerance. Similarly, for patients experiencing prolonged sedation effects, our data support the strategy of initiating RMT only after the patient is fully awake and cooperative, ensuring safety and meaningful engagement. The need for individualised assessment aligns with recommendations by (Mariano et al. 2022) for using consistent daily criteria to evaluate patient status. However, our study extends this concept by highlighting the critical importance of dynamic, daily adjustment of rehabilitation goals and intensity based on these assessments—a key operational insight derived from our local implementation experience. Therefore, a central recommendation arising from our study is the implementation of responsive, patient‐centred RMT protocols. Healthcare professionals should be empowered to continuously modify training based on real‐time patient assessments. This adaptive strategy, directly informed by our findings, is essential for enhancing patient compliance and participation, ultimately overcoming the significant physiological barriers identified in this research.

## 5 Limitations

This study has several limitations. The single‐centre design limits the generalizability of the findings, as they may not reflect the culture and characteristics of other ICUs—a common limitation in qualitative research (Liew et al. 2021). Additionally, participants were recruited from a single ICU, making it difficult to generalise the results to other settings, where distinct cultures, resources and care delivery processes may influence perceptions of barriers and facilitators to RMT. Finally, the rigorous approach used to gain an in‐depth understanding of the issues can serve as a model for other quality improvement projects.

## 6 Conclusion

RMT is increasingly recognised in ICUs and has demonstrated significant benefits for patients after MV withdrawal. However, despite advancements in RMT knowledge, its clinical implementation lags. This qualitative study explores the perceptions of healthcare professionals and patients regarding RMT, identifying both barriers and facilitators. Notably, addressing barriers can simultaneously enhance facilitators. The findings provide actionable insights for future quality improvement projects or research, aiming to integrate RMT seamlessly into nursing routines, not only in ICUs but also in other clinical settings where RMT is applicable. RMT implementation is a multifaceted endeavour, requiring a dynamic and adaptable approach.

## Author Contributions

Study design: Jianing Yin, Xiaomin Guan and Lan Chen. Data collection: Qi Zhang and Xiaodan Zhang. Data analysis: Jianing Yin, Xiaomin Guan, Qi Zhang and Yan Shen. Manuscript writing: Jianing Yin, Xiaomin Guan and Xiaodan Zhang. Supervision, Writing – review and editing: Yan Shen, Lan Chen.

## Funding

This work has been supported by: Shanghai Hospital Development Centre Foundation (SHDC22023233).

## Ethics Statement

The study was approved by the Institutions Review Board (No.【2023】472).

## Conflicts of Interest

The authors declare no conflicts of interest.

## Data Availability

The data that support the findings of this study are available from the corresponding author upon reasonable request.
